# Application of Thinned-Skull Cranial Window to Mouse Cerebral Blood Flow Imaging Using Optical Microangiography

**DOI:** 10.1371/journal.pone.0113658

**Published:** 2014-11-26

**Authors:** Yuandong Li, Utku Baran, Ruikang K. Wang

**Affiliations:** 1 Department of Bioengineering, University of Washington, Seattle, Washington, United States of America; 2 Department of Electrical Engineering, University of Washington, Seattle, Washington, United States of America; Singapore Immunology Network, Singapore

## Abstract

*In vivo* imaging of mouse brain vasculature typically requires applying skull window opening techniques: open-skull cranial window or thinned-skull cranial window. We report non-invasive 3D *in vivo* cerebral blood flow imaging of C57/BL mouse by the use of ultra-high sensitive optical microangiography (UHS-OMAG) and Doppler optical microangiography (DOMAG) techniques to evaluate two cranial window types based on their procedures and ability to visualize surface pial vessel dynamics. Application of the thinned-skull technique is found to be effective in achieving high quality images for pial vessels for short-term imaging, and has advantages over the open-skull technique in available imaging area, surgical efficiency, and cerebral environment preservation. In summary, thinned-skull cranial window serves as a promising tool in studying hemodynamics in pial microvasculature using OMAG or other OCT blood flow imaging modalities.

## Introduction

The *in vivo* imaging of brain microstructure and microcirculation has been a valuable approach to understand vascular function under normal and pathologic conditions. Within the complex vascular network, the pial vessels are intracranial vessels on the surface of the brain within leptomeninges layer of the cortex, which give rise to smaller penetrating arterioles that supply blood to the corresponding region of cerebral cortex [1]. The pial vessels form an effective collateral network, which comprises distal anastomoses from branches of anterior, middle, and posterior cerebral arteries [2]. Such network is responsible for the redistribution of flow when there is a constriction or an occlusion at one of the arteries/arterioles, serving as a vital element in rescuing an ischemic region [3]. In experimental stroke research, the examination of pial vessels on the brain cortex during acute injury and chronic recovery provides critical information for studying the mechanisms underlying ischemic stroke [4].

A variety of non-invasive *in vivo* imaging techniques have been developed to explore the cerebral pial microvasculature. Two-photon microscopy provides structural imaging from fluorophores inside living tissue that are excited by simultaneous absorption of two photons [5] [6]. It has been widely applied for neuronal connectivity investigation [7] [8], as well as blood flow activity by labeling red blood cells (RBC) [9]. Although it provides great resolution (∼1 µm), it is typically limited with a shallow imaging depth (∼300 µm) and small field of view (up to ∼300×300 µm^2^) [10], which make it difficult to acquire images covering a large area under time-sensitive experiments such as the middle cerebral artery occlusion (MCAO) model in mice. Moreover, laser Doppler imaging (LDI) [11] and laser speckle imaging [12] are alternative imaging techniques which allow *in vivo* measurement of microcirculatory flow by applying the Doppler effect or calculating the speckle contrast, respectively. Although these approaches have proved their ability in visualizing relative changes in blood perfusion in the cortex with a large field of view, they are limited in image resolution to resolve detailed vessels and cannot provide absolute velocity of blood flow.

Optical coherence tomography (OCT) is another non-invasive imaging technique capable of producing large-scale (millimeters) cross-sectional morphological views of microstructure *in vivo* with a micron-level resolution [13] [14]. Regardless of its great potential in structural imaging, traditional OCT experiences difficulties in providing blood vessel information. Fortunately, a newly developed technique called optical microangiography (OMAG) has the ability to analyze both intensity and phase information embedded in the OCT spectral interferogram to produce 3D blood perfusion map *in vivo* [15] [16] [17]. Additionally, ultra-high-sensitive OMAG (UHS-OMAG), an improved variation of OMAG technique, has been recently proposed to achieve better sensitivity of capillary vessel imaging [18] [19]. In the past few years, UHS-OMAG has been successfully used in studying the mouse cerebral microvasculature and its response to systemic hypoxia, normoxia, and hyperoxia [20]. By combining Doppler principle and OMAG, Doppler OMAG (DOMAG) [21] has also been developed and used in dermatology applications to image capillary morphology in human finger cuticle [22] [23] and to study microvascular response to inflammation induced by tape stripping on human skin *in vivo* [24]. Based on the previous literatures, a combination of UHS-OMAG and DOMAG is a powerful tool to investigate pial vessel structure and perfusion status on the brain surface.

Tissue scattering is one of the greatest obstacles faced by all optical imaging modalities [25]. The resolution, imaging depths and image quality are strongly affected by the light absorption and high scattering at the cranial tissue during brain imaging. Therefore, two distinct types of cranial window techniques are developed to overcome these problems: the traditional cranial window, also known as open-skull window technique, in which part of the skull is removed and replaced with a glass coverslip [26] [27] [28]; thinned-skull technique, in which a selected area of the skull is thinned down to a thickness of ∼15–50 µm [29] [30] [31]. Although both methods have shown their ability to visualize structural and functional changes in brain vasculature, the invasive nature of open-skull window technique is more likely to create alterations in the basal condition of cerebral blood flow (CBF), which makes it difficult to interpret experimental data [10].

To our knowledge, there has not been any established preference in literature between a thinned-skull and an open-skull cranial window for mouse CBF imaging. In this paper, using UHS-OMAG and DOMAG imaging modalities, we compared these two cranial window techniques concerning surgical invasiveness and efficiency, as well as their ability to reveal pial microvasculature on mouse brain. Moreover, we improved the thinned-skull techniques by combining protocols with thinning procedure [31] and polishing procedure [30] together, so that a large imaging window with a smoother surface was created to greatly reduce the cranial tissue scattering. As a result, the application of cranial windows achieved higher resolution of pial vessels compare to the intact skull case, and the thinned-skull technique had advantages over the open-skull window in the efficiency of surgical procedures and the available imaging area. More importantly, thinned-skull technique is found to apply minimal changes to the basal condition in the cerebral blood flows, and therefore, is recommended in studying hemodynamics in pial vessels during short-term stroke studies.

## System and Methods

### Animal Models

All experimental animal procedures performed in this study were approved by the Institute of Animal care and Use Committee (IACUC) of the University of Washington (Protocol number: 4262-01). All procedures were performed under anesthesia and all efforts were made to minimize suffering.

**Figure 1:**
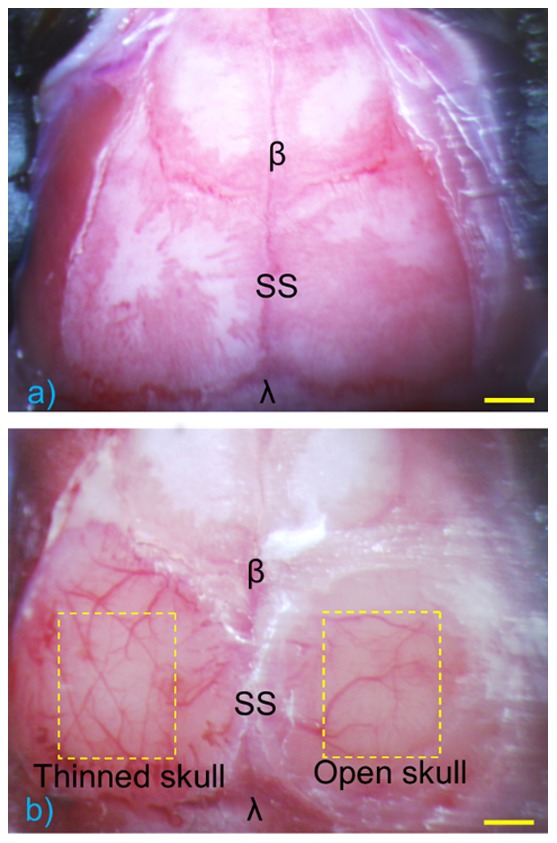
Microscopic images of skull before and after cranial windows comparison. (a) Baseline of the skull where cranial windows are applied later, showing bregma lambda, and sagittal suture. (b) Left: Thinned-skull window with 4×4 mm available imaging area. Right: Open-skull cranial window with 3×3 mm available imaging area. The left cortex, thinned to 20∼30 µm, is comparably transparent under microscope compare to the right side of the cortex with open-skull window. β = bregma, λ = lambda, SS = sagittal suture. Scale bar represents 1 mm.

Total 7 mice were used in the study. Data from 2 of them was excluded from the presented results due to severe subdural hematoma occurred during thinned-skull cranial window preparation. Five mice were subjected to microscopic imaging, OMAG baseline and OMAG cranial windows, generating 5 sets of the OMAG results for resolution comparison and vessel density analysis. Three-month-old C57/BL6 mice weighing 23–25 g were obtained from Charles River Laboratories. The mouse was deeply anesthetized using 1.5–2% isoflurane (0.2 L/min O2, 0.8 L/min air) through a nose cone and placed on a stereotaxic frame and supported by a heating pad to maintain body temperature during the entire experiment process. After the disappearance of the toe pinching response, the skin and periosteum was removed to expose the skull, and a microscopic image was taken (Figure 1a). The skull was then subjected to a baseline imaging by OMAG (see Imaging Protocol). Drops of saline were applied to the skull constantly to keep wet during imaging process. After the OMAG baseline was taken, the two types of cranial window procedures were performed on each side of parietal bone (see Cranial Window Technique). Brain cortex transparency produced by the application of cranial windows is demonstrated under microscope (Fig. 1b), and the two cranial windows were imaged by OMAG with the same imaging protocol used for the baseline imaging (see Imaging Protocol).

### Cranial Window Techniques

Before starting cranial window procedures, dexamethasone sodium phosphate (0.02 ml at 4 mg/ml; ∼2 ug/g dexamethasone) was administered by subcutaneous injection to prevent cerebral edema. The skull surface was cleaned and dried with sterile saline and cotton swaps. Then, a drop of 1% Xylocaine (Lidocaine 1%+epinephrine 1∶100,000 solution) was applied directly onto the skull to minimize the bleeding of the skull.

In preparation of a thinned-skull window, a selected area (4×4 mm) on the left parietal bone ∼1.5 mm posterior and lateral to bregma was thinned down to 20∼30 µm. The skull was thinned by a high-speed surgical hand drill (Foredom Electric Co., Ethel, CT, USA) with a round carbide bur (0.75 mm) followed by a green stone bur (Shofu Dental Corp., San Marcos, CA, USA). The skull was constantly wetted with sterile saline to remove the bone dust and to prevent overheating. When the thickness of the skull reaches ∼30 µm with the pial vessels clearly visible through wetted bone, a polishing step was followed. The skull was sequentially polished with size 3F grit followed by size 4F grit (Convington Engineering, Redlands, CA, USA). A slurry of grid and saline was agitated with a custom made silicone coated drill bit. The polished skull was finally washed with sterile saline and the remaining bone dust were picked up by saline-soaked gelfoam.

For an open-skull cranial window, a circular piece of skull (∼4×4 mm) at the region of interest ∼1.5 mm posterior and lateral to bregma was removed and replaced with a round coverglass (Thermo Scientific, Waltham, MA, USA). To do so, a circular groove was first thinned with a shaft drill (Foredom Electric Co., Ethel, CT, USA). The groove was drilled slowly and saline was applied regularly to avoid heating. In the end, the central island of skull bone was lifted and removed with the help of saline, replacing with a circular coverglass sealed to the skull by cyanoacrylate glue, which brought the available imaging area to ∼3×3 mm.

### System setup

A fiber-based SD-OCT system was used for the experiments [22]. A superluminescent diode (Thorlabs Inc., Newton, NJ, USA) was used as the light source, which had a central wavelength of 1340 nm and a bandwidth of 110 nm that provided a ∼7 µm axial resolution in the air (∼5 µm in tissue if the refractive index of tissue is taken as 1.35). In the sample arm, a 10× scan lens (Thorlabs Inc., Newton, NJ, USA) was used to achieve a ∼10 µm lateral resolution with 0.12 mm depth of view. The output light from the interferometer was routed to a home-built spectrometer, which had a designed spectral resolution of ∼0.141 nm that provided a detectable depth range of ∼3 mm on each side of the zero delay line. The line rate of the linescan camera (Goodrich Inc., Princeton, NJ, USA) was 92 kHz. The system had a measured dynamic range of 105 dB with the light power of 3.5 mW at sample surface. The operations for probe beam scanning, data acquisition, data storage and hand-shaking between them were controlled by a custom software package written in Labview.

### Imaging Protocol

To visualize the volumetric microvasculature, a unique UHS-OMAG scanning protocol was applied [19]. Briefly, microvasculature down to capillary level was visiualized by separating structural tissue from dynamic scatters (e.g., moving red blood cells within patent vessels) using an ED-based clutter filtering algorithm [32]. In this protocol, each B-frame consisted 400 A-lines covering a distance of ∼2.0 mm. The imaging rate was 180 fps. In the slow axis (C-scan), a total number of 2000 B-frames with 5 repetitions in each location were performed, also covering a distance of ∼2.0 mm. Hence the data cube of each 3D image was composed of 1024 by 400 by 400 (z-x-y) voxels, which took ∼11 s to acquire.

After each UHS-OMAG scan, DOMAG measurement was performed, covering the same area to show the axial velocity map of CBF [22]. In brief, phase-resolved technique [33] [34] used to calculate the axial flow velocity of the RBC is shown,
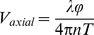
(1)where λ is the center wavelength of light source, n is the tissue refractive index, and φ and *T* are the phase difference and time interval between adjacent A-lines, respectively. The maximal and minimal detectable velocities are determined by the time interval *T*, the π-ambiguity and the system phase noise level [33]. Each B-scan in DOMAG protocol contained 10000 A-lines by acquiring 25 A-lines at each 400 discrete steps. In the elevational direction, there were 300 discrete points, i.e., 300 B scans. Three A-lines were skipped during Doppler processing to increase the time interval, T, between processed A-lines, and frame rate was set to 6 fps which gave an axial velocity range of ±6.1 mm/s. The data cube of each 3D image (C scan) was composed of 1024 by 400 by 300 (z-x-y) voxels, which took ∼100 s to acquire with 6 fps imaging speed. Details of this imaging protocol can be found in [18] [22].

To acquire the CBF images over a large area of the cortex, the scan was repeated to create a mosaic image. During the entire experiment sequence, this imaging protocol was firstly applied to the intact skull. After creating the cranial windows, same imaging protocol was repeated over the same area by keeping the discrepancies between those two cases minimum in terms of the focus of probe beam and the orientation of sample.

## Results

**Figure 2:**
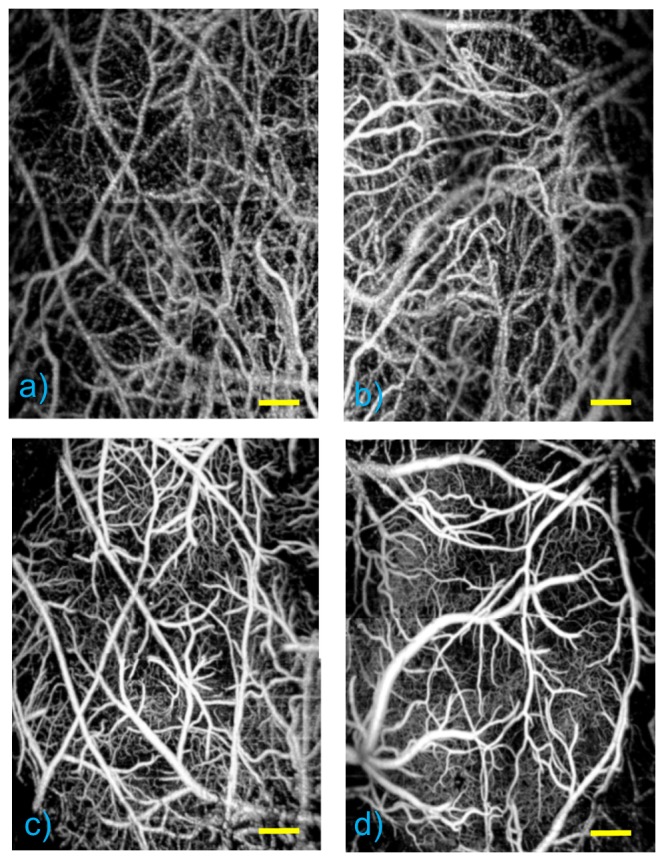
UHS-OMAG MIP view of microcirculation network up to ∼1 mm depth. (a–b) With intact skull (baseline). (c) With thinned-skull cranial window. (d) With open-skull cranial window. Scale bar represents 0.4 mm.

The volumetric UHS-OMAG maximum intensity projection (MIP) result of capillary network in the pial region of mouse brain is presented in Figure 2, corresponding to the yellow dashed area in the microscopic image (Fig. 1b). Surface vessel dynamics play an important role in CBF regulation. Hence, the microcirculation at up to 200 µm depth is targeted, which is arranged to cover the depth of focus of the lens. The final image, representing the CBF over the mouse cortex in the area of interest, is obtained by stitching and cropping 4 images together, for each side of the parietal bone. This procedure is applied both to baseline with intact skull (Fig. 2a–b) and to two cranial window cases (Fig. 2c–d) to compare the visible vessel density changes regarding to two different cranial window methods.

**Figure 3:**
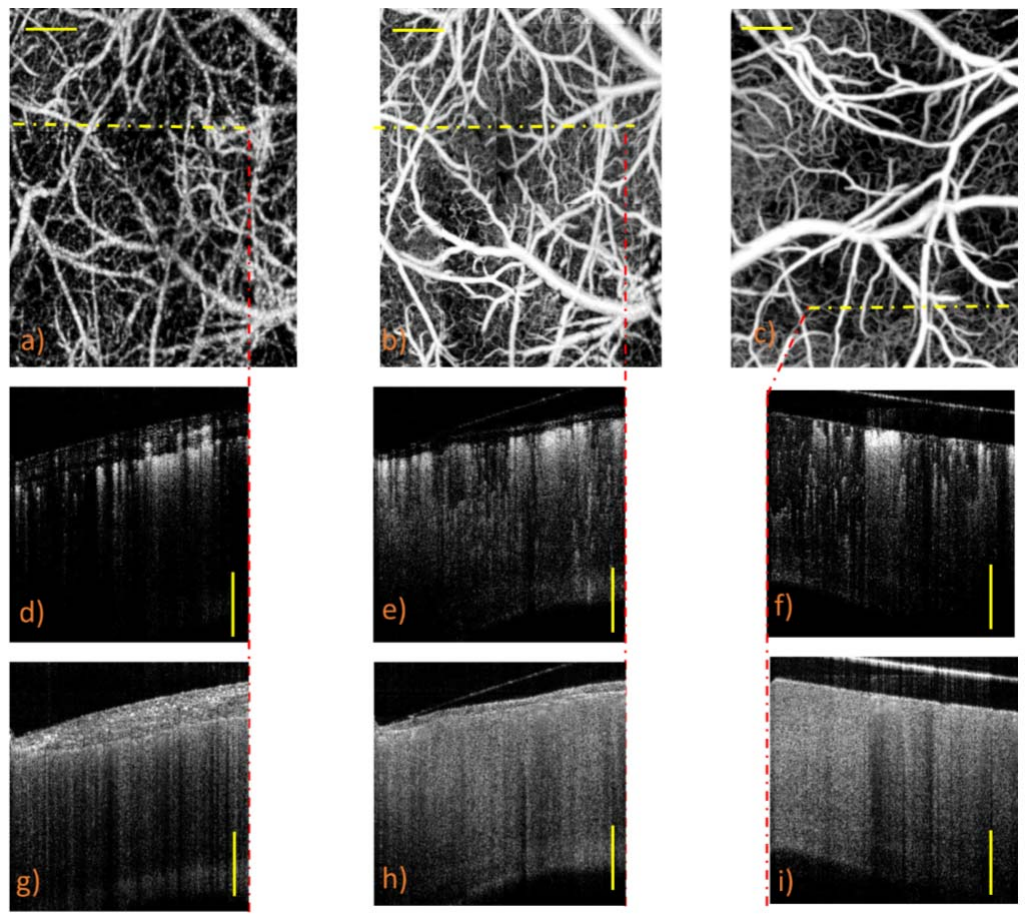
MIP view of UHS-OMAG images. (a) Before thinned-skull (baseline). (b) After thinned-skull cranial window procedure. (c) After open-skull cranial window procedure. (d–f) The blood perfusion images of the cross-sections located at dotted yellow lines. (g–i) The structural images of the same cross-sections. Scale bar is 0.3 mm.

To identify the location of the blood vessels and to determine the remaining thickness of the thinned-skull, UHS-OMAG images are merged with structural OCT images for both before and after cranial windows. Figure 3 presents maximum projection view of UHS-OMAG images of intact skull (Fig. 3a), thinned-skull (Fig. 3b) and open-skull cranial windows (Fig. 3c). Moreover, the cross sectional blood perfusion and structural images corresponding to the positions marked by the dash yellow lines are presented in Fig. 3(d–f) and Fig. 3(g–i), respectively. From the cross sectional structural images remaining thickness of the thinned-skull is estimated ∼20–30 µm.

**Figure 4:**
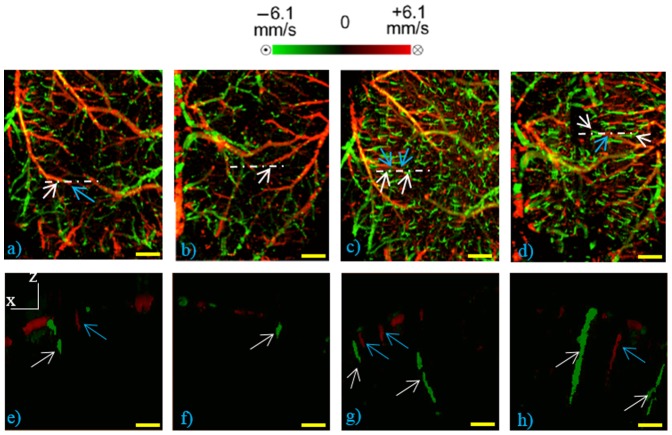
DOMAG MIP view of microcirculation network at 50–450 µm depth. (a–b) Shows baseline before the application of cranial windows to intact skull. (c) With thinned-skull cranial window (d) With open-skull cranial window. The white dashed lines represent the location of the cross sectional views (along x–z planes) shown in (e–h). White arrows point the diving arterioles and blue arrows point the rising venules. Scale bar represents 0.5 mm in (a–d) and represent 0.15 mm in (e–h).

Furthermore, the same area is imaged using DOMAG protocol to obtain CBF axial velocity mapping. The bidirectional en face MIP images in Figure 4 shows the diving and rising vessels as green and red lines, respectively, where RBC axial velocity information is coded with a color bar in a range of ±6.1 mm/s. The diving arterioles and rising venules appear as green and red isolated spots. This is because their flow directions are mainly parallel to the beam axis, giving their axial velocity large enough to be detected at this range. However, the surface vessels connected to them, whose flow nearly perpendicularly to the probe beam, escaped the detection due to their small axial velocity. Few out-of-range flows are also observed as phase-wrapped signals, seen as yellow color (combination of red and green).

Figure 4 (a–b) demonstrate the CBF axial velocity mapping of intact-skull (baseline) case whereas Fig. 4(c–d) show the result of after applying two distinct cranial windows to each side of the mouse head. Moreover, cross sectional views of the areas corresponding to white dashed lines are shown in Fig. 4(e–f) for each case. For the intact-skull case, although the bigger vessels can be visualized, the diving arterioles or rising venules are harder to detect as seen in Fig. 4(e–f). On the other hand, Fig. 4(g–h) shows that both type of cranial windows provide better visualization of the diving arterioles and rising venules. Furthermore, similar to the UHS-OMAG case shown in Fig. 2–3, thinned-skull cranial window provides larger area with a better visibility.

**Figure 5:**
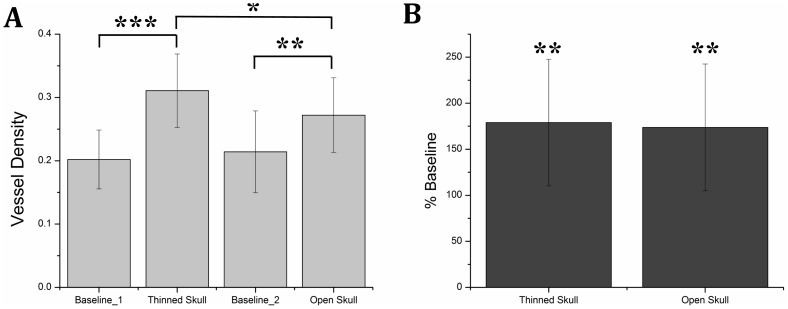
Vessel density comparison and total blood flow quantification. (a) Comparison of detectable vessel density (n = 5). Baseline_1 refers to basal condition before the thinned skull cranial window and baseline_2 refers to basal condition before the open skull cranial window. (b) Comparison of total blood flow among penetrating arterioles in thinned skull and open skull with respect to intact skull case. (n = 6). There is no significant differences between thinned-skull case and open-skull case. Data represent mean±s.e.m. ***P<0.01, **P<0.05, and *P<0.3 significantly different data sets (paired t-test).

In order to quantify the change in the quality of the blood perfusion image, the vessel density of images from identical areas for each case was calculated using a segmentation algorithm [35]. First, the identical areas were selected from the whole images and registered using the function, *imregister*, available in MATLAB®. Then, registered images were segmented for the blood vessels by creating a binary black and white image with an adaptive threshold technique specifically designed for OMAG blood flow images [36]. In this technique, a low-pass filter was used to minimize the elements that were smaller than a specific radius size. After that, a global threshold was used to set to zero all the pixels below a certain threshold. Finally, a local adaptive threshold was implemented to binarize the image based on the mean pixel value within a predefined window size. The vessel density was calculated by dividing the number of ones with the total pixel number. Figure 5a shows the mean visible vessel densities of 5 samples for each case. The results indicate that mean values of visible vessel densities of baselines (intact skull) are similar for both left and right sides of mouse brain. Moreover, thinned-skull window provides 60% more details compare to baseline case and 15% more details than the open-skull case, in average.

The overall blood flow changes among the penetrating arterioles in the imaged areas were calculated for each case using the method described in [22]. Figure 5b shows the mean relative differences between the cranial window cases with the corresponding baselines. Accordingly, both thinned-skull and open-skull cranial windows enable higher image quality and deeper light penetration depth, which in return give more accurate total blood flow calculation.

## Discussion

In our experiments, we demonstrated the feasibility of visualizing and quantifying pial vessels using OMAG techniques *in vivo*. Our results show that the quality of the cerebral microvasculature imagining can be significantly affected by the thick skull layer for OMAG, as well as other optical signal intensity dependent OCT imaging. Even though keeping skull intact is the ideal non-invasive model for *in vivo* brain imaging, a good choice of cranial window methods is desired to reduce skull tissue scattering effect when higher quality images are needed. Therefore, we investigated two common types of cranial window procedures, open-skull and thinned-skull techniques, in regards to their imaging quality outcomes and procedure practicality. From our results, OMAG imaging through cranial windows increased the signal intensity and light penetration depth as compared to normal intact skull at the signal intensity profile (Figure 3). Additionally, the details of penetrating arterioles are significantly preserved in both cranial window cases than in the normal intact skull, which in return give more accurate total blood flow calculation.

When comparing the differences between two cranial window types, we found that the available imaging area these two methods differs from ∼3×3 mm in open-skull case to ∼4×4 mm in thinned-skull case, which is crucial when quantifying blood vessels or investigating multiple anastomoses between major cerebral arteries during a stroke study. Additionally, during open-skull cranial window procedure, excess glue around the window edge may leak inside the window, which further deteriorates the available imaging area. Overall, OMAG imaging with a thinned-skull window provides a larger available imaging area, while permitting a similar penetration depth with an open-skull window. Another advantage of a thinned-skull over an open-skull window is the procedure efficiency. From our experience, a thinned-skull procedure consumes less surgical time (15 min) compare to an open-skull cranial window method (30 min). Since the thinned-skull method does not require a craniotomy, complications are less during the surgery, such as excessive bleeding or swollen of the dura mater, which requires more training and practice effort to prevent. We further compared the invasiveness of these two window opening techniques by assessing the effects on vascular density and vessel loss within the imaging windows. The data shows that, in average, the thinned-skull method greatly reduces the invasiveness of the surgical procedure and preserves 15% more details in pial vessel imaging than the open-skull case. One of the reasonable explanation of loss of vessels in the open-skull case is that while employing a craniotomy on the skull, we encountered some bleeding owing to tearing of the vascular plexus in the trabecular section of the cranial bone. Under ideal circumstances dura should not bleed. However, in our experience, since dura is attached to the inner table of the cranium some superficial capillaries might tear during removal of cranial bone and small focal bleeding occur occasionally. Hence, these likely imperfections during the procedure may deteriorate the imaging quality and detailed vasculature network in an open-skull window. To summarize, in order to achieve higher OMAG quality while preserving a close-to-normal cerebral condition *in vivo*, a thinned-skull cranial window serves as a great compromise between craniotomy and intact skull.

Nevertheless, there are limitations when using the thinned-skull technique. First of all, the technique still brings injury to the skull, and we encounter some sub-dural hemorrhage due to vibration of drilling and interfere from time to time, in which case a new animal model has to be obtained for best OMAG turnout. In a global scale of vessel visualization which does not require detailed blood flow information, a normal intact skull is still a preferred non-invasive method to apply. Secondly, since the remaining thinned skull lacks vasculature, it starts to become opaque within hours, and thinning needs to be repeated if more imaging sessions are followed days after [27] [28] [29] [30]. Therefore, for chronic study, an open-skull cranial window or a variation of the thinned-skull technique, re-inforced thinned skull, needs to be applied for long-term imaging purpose [30]. Yet, because of the efficiency and less invasive nature of the thinned skull technique, additional procedures (such as MCAO etc.) can be performed right after; whereas after open-skull cranial window, it is suggested to give the animal a 24 hr recovery period before performing any surgeries [27]. Therefore, the thinned-skull model developed in this paper is suitable for immediate imaging to compare effect of additional surgeries. In the duration of a short-term imaging, a plastic food wrap can be placed on the wetted window surface to avoid the thinned skull becoming opaque, and to preserve clarity during the imaging sessions.

In this study, we made the effort to keep all the crucial parameters, such as the focus of the probe beam and positioning and orientation of the target to be the same among different samples. The image registration steps performed in the post-processing are crucial to have a reliable quantitative comparison between the images of the same area for various cases.

## Conclusions

In this study, we evaluated two cranial window variations, thinned-skull and open-skull methods, for cerebral blood flow imaging using OMAG. We applied both techniques on the skull of the same animal and compared them with the normal intact skull condition in regards to procedure practicality and their ability to reveal the pial microvasculature on mouse brain. The results proved that creating cranial windows reduced the effect of skull scattering and substantially increased the signal penetration depths for OMAG imaging. Choosing between two cranial window types, the thinned-skull technique has advantages in preserving a better cerebral environment, which allows for a more accurate measurement and quantification of brain vasculature. Our improved thinned-skull method serves as a promising model for *in vivo* cerebral vessel tracking and flow calculation during short-term study using OMAG and other intensity-based OCT imaging modalities, which can be applied to study immediate CBF response upon MCAO stroke or traumatic brain injury.
